# Das Hepatitis-E-Virus – ein zoonotisches Virus: Verbreitung, Übertragungswege und Bedeutung für die Lebensmittelsicherheit

**DOI:** 10.1007/s00103-021-03476-w

**Published:** 2022-01-04

**Authors:** Reimar Johne, Nadine Althof, Karsten Nöckler, Alexander Falkenhagen

**Affiliations:** Bundesinstitut für Risikobewertung, Max-Dohrn-Str. 8–10, 10589 Berlin, Deutschland

**Keywords:** Hepatitis E, Zoonose, Schwein, Fleischprodukte, Virus-Inaktivierung, Hepatitis E, Zoonosis, Pig, Meat products, Virus inactivation

## Abstract

Das Hepatitis-E-Virus (HEV) ist ein Erreger einer akuten Hepatitis beim Menschen. Darüber hinaus treten zunehmend auch chronische Infektionen mit fataler Leberzirrhose bei immunsupprimierten Transplantationspatienten auf. Die Zahl der gemeldeten Hepatitis-E-Fälle in Deutschland hat in den vergangenen Jahren stark zugenommen. Hier kommt vor allem der Genotyp 3 vor, der zoonotisch von Tieren auf den Menschen übertragen werden kann. Haus- und Wildschweine, die ohne die Ausbildung klinischer Symptome infiziert werden, stellen das Hauptreservoir dar. In diesem Artikel werden die Verbreitung von HEV in Tieren in Deutschland, mögliche Übertragungswege des Virus und insbesondere die Bedeutung von Lebensmitteln bei der Übertragung anhand der aktuellen wissenschaftlichen Literatur dargestellt.

HEV ist in Haus- und Wildschweinen in Deutschland stark verbreitet und wird hauptsächlich über direkten Kontakt oder den Verzehr von Lebensmitteln, die aus diesen Tieren hergestellt wurden, auf den Menschen übertragen. Beim HEV-RNA-Nachweis in spezifischen Lebensmitteln bleibt allerdings oft unklar, ob das enthaltene Virus noch infektiös ist oder durch die Herstellungsbedingungen inaktiviert wurde. Neuere Studien weisen auf eine hohe Stabilität des HEV unter verschiedenen physikochemischen Bedingungen hin, wohingegen eine Inaktivierung unter anderem durch Erhitzung erreicht wird. Generell wird deshalb ein ausreichendes Erhitzen von Schweinefleisch und -leber vor dem Verzehr empfohlen und für Risikogruppen zusätzlich der Verzicht auf den Verzehr kurzgereifter Rohwürste.

Weitere Forschungen sind nötig, um relevante Risikolebensmittel zu identifizieren, alternative Übertragungswege zu untersuchen und effiziente Maßnahmen zu entwickeln, die eine zoonotische Virusübertragung zukünftig verringern oder vermeiden.

## Abstract (en)

The hepatitis E virus (HEV) is an etiological agent of acute hepatitis in humans. In addition, chronic infections resulting in fatal liver cirrhosis currently emerge in immunosuppressed transplant patients. The number of notified hepatitis E cases in Germany has steeply increased in recent years. Here, genotype 3, which can be zoonotically transmitted from animals to humans, is predominant. The main reservoirs are pigs and wild boars, which show no signs of infection. In this article, the distribution of HEV in animals in Germany, possible transmission pathways, and especially the importance of food as a transmission vehicle are presented based on the current scientific literature.

HEV is widely spread among domestic pigs and wild boars in Germany and the virus is mainly transmitted by direct contact or by consumption of food produced from those animals. However, if HEV RNA is detected in specific food it is often unclear whether the contained virus is still infectious or inactivated by the conditions during production. Recent studies indicate a high stability of HEV against different physicochemical conditions, whereas – among others – the virus can be efficiently inactivated by heating. Therefore, proper heating of pork meat and liver prior to consumption in general is recommended. For risk groups, avoiding shortly cured raw sausages is an additional suggestion.

Further research is necessary to identify relevant risk food products, to investigate alternative transmission pathways, and to develop efficient measures in order to reduce or prevent zoonotic transmissions of the virus in future.

## Einleitung

Infektionen mit dem Hepatitis-E-Virus (HEV) können beim Menschen zu einer akuten Leberentzündung führen. In den meisten Fällen verläuft die Erkrankung moderat und selbstlimitierend; die Mortalitätsrate wird mit 0,5–4 % angegeben. Schwere Verläufe mit Todesfällen kommen vor allem bei Risikogruppen vor. Bei Infektionen mit dem HEV-Genotyp 1 zeigen vor allem schwangere Frauen schwere Erkrankungsverläufe, während bei Personen mit Lebervorschädigungen häufig schwere Verläufe bei HEV-Genotyp 3‑Infektionen vorkommen. Dieser Genotyp löst bei immunsupprimierten Personen, vor allem bei Transplantationspatienten, auch chronische HEV-Infektionen aus, die oft zu einer fatalen Leberzirrhose führen. Neben der Manifestation in der Leber wurden auch extrahepatische Krankheitsverläufe beschrieben, die vor allem durch neurologische Symptome gekennzeichnet sind, wie sie beim Guillain-Barré-Syndrom zu finden sind oder durch Meningoenzephalitis oder Neuritis [1].

Infektionen mit HEV treten weltweit auf. In Deutschland ist die Zahl der gemeldeten Hepatitis-E-Fälle in den vergangenen Jahren deutlich gestiegen. Waren es im Jahr 2009 noch 109 registrierte Fälle, wurden in 2019 insgesamt 3728 Fälle gemeldet [2]. Der Anstieg wird vor allem auf eine höhere Aufmerksamkeit der Ärzte für diese Erkrankung und das Vorhandensein besserer diagnostischer Tests zurückgeführt. Neben diesen klinisch auffälligen Erkrankungsverläufen scheint eine HEV-Infektion jedoch auch häufig mit nur milden Symptomen einherzugehen oder sogar asymptomatisch zu bleiben. Serologische Untersuchungen zeigen, dass im Jahr 2010 etwa 15,3 % der deutschen Bevölkerung HEV-spezifische IgG-Antikörper besaßen, die auf eine vergangene Infektion hinweisen [3].

Das HEV ist ein etwa 30–40 nm großes Viruspartikel, das ein Genom aus Einzelstrang-RNA mit positiver Polarität besitzt. Während es über den Darmtrakt als unbehülltes Virus ausgeschieden wird, findet man im Serum von Patienten und im Überstand infizierter Zellkulturen vor allem Partikel, die eine zusätzliche Lipidhülle besitzen. HEV wird in die Familie *Hepeviridae*, Genus *Orthohepevirus* eingeordnet, in dem Stämme der Spezies *Orthohepevirus A* für den Menschen die wichtigste Rolle spielen (Tab. 1). Innerhalb dieser Spezies wurden bisher 8 unterschiedliche Genotypen beschrieben, die sich bezüglich ihres Wirtsspektrums und ihrer Übertragungswege teilweise deutlich unterscheiden [4].

**Table 1:** 

Spezies	Genotyp	Hauptsächlicher Wirt	Hauptsächliche geografische Verbreitung	Hauptsächlicher Übertragungsweg zum Menschen
*Orthohepevirus A*	1	Mensch	Asien, Afrika	Verunreinigtes Trinkwasser
	2	Mensch	Mittelamerika, Afrika	Verunreinigtes Trinkwasser
	3	Mensch, Schwein, Wildschwein	Weltweit	Verzehr von Fleischprodukten
	4	Mensch, Schwein, Wildschwein	Südostasien	Verzehr von Fleischprodukten
	5	Wildschwein	Japan	–
	6	Wildschwein	Japan	–
	7	Dromedar, Mensch	Mittlerer Osten	Unklar
	8	Trampeltier	Südostasien	–
*Orthohepevirus B*	n. d.	Verschiedene Vogelarten	Weltweit	–
*Orthohepevirus C*	n. d.	Verschiedene Rattenarten, Frettchen, Nerz, Mensch	Weltweit	Unklar
*Orthohepevirus D*	n. d.	Verschiedene Fledermausarten	Weltweit	–

*n.* *d*. nicht definiert, – keine Zoonose

Die Genotypen 1 und 2 infizieren ausschließlich den Menschen und werden vor allem über kontaminiertes Trinkwasser übertragen. Dies führt in Entwicklungsländern häufig zu großen Krankheitsausbrüchen. Bei der Infektion von Schwangeren sind auch vertikale Übertragungen dieser Genotypen möglich [4]. Demgegenüber sind die Genotypen 3 und 4 zoonotisch und haben ihr Hauptreservoir in Haus- und Wildschwein, von denen sie vor allem über den Verzehr von Fleischprodukten auf den Menschen übertragen werden können. Diese Genotypen sind vorrangig in Industrieländern weitverbreitet und führen hier zu sporadischen Hepatitis-E-Fällen. Neben dem zoonotischen Übertragungsweg können diese Genotypen auch parenteral über Blut und Blutprodukte übertragen werden [4]. Aus der Virusspezies *Orthohepevirus A* wurde weiterhin der Genotyp 7 beim Menschen gefunden, der sein Reservoir vor allem in Dromedaren hat und im Mittleren Osten vorkommt. Als weiteres humanpathogenes Virus wurde kürzlich das Ratten-HEV identifiziert, das besonders in Ratten verbreitet ist und zur Spezies *Orthohepevirus C* gehört [1, 4]. Tab. 1 zeigt einen Überblick über die Hepevirusspezies, Genotypen und deren wichtigste Charakteristika.

In Deutschland und in vielen anderen europäischen Ländern werden hauptsächlich Infektionen mit dem HEV-Genotyp 3 mit den Subtypen 3c, 3e und 3f gefunden [5]. Diese Subtypen werden hier gleichermaßen auch in Schweinen, Wildschweinen und anderen Tierarten nachgewiesen, weshalb ein zoonotischer Übertragungsweg auf den Menschen über den Kontakt mit den Tieren oder über den Verzehr von Fleischprodukten wahrscheinlich ist. In diesem Artikel sollen die Übertragungswege des HEV in Deutschland und die daraus resultierenden Konsequenzen für die Lebensmittelsicherheit genauer betrachtet werden. Hierzu wird zunächst die aktuelle Literatur zur Verbreitung des Virus – im Speziellen des Genotyps 3 – in Tieren, zu prinzipiell möglichen Übertragungswegen und zu Untersuchungen an Lebensmitteln vorgestellt, um anschließend sowohl Schlussfolgerungen für die Verhinderung einer Virusübertragung durch Lebensmittel zu ziehen als auch Forschungsbedarf zur Klärung offener Fragen aufzuzeigen.

## Verbreitung des HEV-Genotyps 3 in Tieren

Zur Verbreitung von HEV-Infektionen in Hausschweinen liegen weltweit zahlreiche Studien vor [6]. Hierbei sind Studien zur HEV-Antikörperprävalenz von solchen zur HEV-RNA-Prävalenz zu unterscheiden. Während Antikörper eine durchgemachte Infektion anzeigen und damit auch weit zurückliegende Infektionen nachweisen können, deuten Virus-RNA-Nachweise auf eine Infektion zum Untersuchungszeitpunkt hin. Tab. 2 fasst die Ergebnisse von Studien zur HEV-Antikörper- und HEV-RNA-Prävalenz in Haus- und Wildschweinen in Deutschland zusammen. Hierbei zeigen sich bei Hausschweinen Antikörperprävalenzen von 42,7–68,6 %, während die Virus-RNA-Prävalenzen zum Zeitpunkt der Schlachtung zwischen 1,0 % und 17,2 % liegen. Die im Vergleich zur Antikörperprävalenz niedrige Virus-RNA-Prävalenz zum Zeitpunkt der Schlachtung spiegelt die Tatsache wider, dass die HEV-Infektion beim Hausschwein vor allem in den ersten Lebensmonaten stattfindet, während zu späteren Zeitpunkten das Virus oft bereits aus dem Körper eliminiert worden ist.

**Table 2:** 

Tierart	Nachweis von HEV-RNA (Zahl positiver/Gesamtzahl untersuchter Tiere)	Nachweis HEV-spezifischer Antikörper (Zahl positiver/Gesamtzahl untersuchter Tiere)	Referenz
Hausschwein	–	534/1072 (49,8 %)	[38]
	–	354/516 (68,6 %)	[39]
	34/251 (13,5 %)	–	[40]
	–	1065/2273 (46,9 %)	[41]
	–	187/438 (42,7 %)	[42]
	1/105 (1,0 %)	–	[35]
	3/120 (2,5 %)	–	[43]
	43/259 (17,2 %)	155/250 (62,0 %)	[44]
Wildschwein	17/189 (5,3 %)	–	[32]
	22/148 (14,9 %)	–	[7]
	48/126 (38,1 %)	32/107 (29,9 %)	[33]
	–	109/330 (33,0 %)	[34]
	18/124 (14,5 %)	–	[35]
	14/134 (10,4 %)	–	[36]
	39/232 (16,8 %)	81/180 (45,0 %)	[10]
	4/104 (3,8 %)	12/104 (11,5 %)	[37]

– nicht untersucht

Auch Wildschweine wurden in zahlreichen Studien auf HEV-Infektionen untersucht (Tab. 2). Hierbei sind für Deutschland Antikörperprävalenzen von 11,5–45,0 % und RNA-Prävalenzen von 3,8–38,1 % berichtet worden. Die hier stärker zutage tretenden Schwankungen lassen sich vor allem mit Unterschieden in verschiedenen Jagdgebieten erklären, wobei auch nah beieinanderliegende Gebiete große Unterschiede in den Nachweisraten aufweisen können [7, 8]. Ein weiterer Unterschied zur Infektion beim Hausschwein scheint die breitere Verteilung von RNA-positiven Tieren in verschiedenen Altersgruppen zu sein. Auch ältere Tiere tragen hier häufiger HEV-RNA. Dies könnte einerseits mit unterschiedlichen Übertragungsmustern bei den frei laufenden Wildschweinen im Vergleich zu den im Stall gehaltenen Hausschweinen erklärt werden. Andererseits gibt es auch Hinweise, dass Wildschweine eventuell häufiger chronische HEV-Infektionen ausbilden können.

Verschiedene Studien liefern Hinweise auf ein breites Spektrum von empfänglichen Tierarten [9]. Kaninchen zeigen hohe Nachweisraten, die auf Infektionen mit einem speziellen Kaninchensubtyp innerhalb des Genotyps 3 zurückzuführen sind. Vereinzelte humane Fälle von Infektionen mit diesem Genotyp weisen das zoonotische Potenzial dieser Virusvariante nach. In den meisten anderen Fällen werden dagegen nur niedrige Nachweisraten sowohl bei Antikörpern als auch bei HEV-RNA gefunden, was gegen eine wichtige Funktion dieser Tierarten als Virusreservoir für humane Infektionen spricht. Vergleichsweise häufig wurden HEV-RNA-Nachweise hingegen bei Wildwiederkäuern berichtet. Eine Studie in Deutschland erbrachte RNA-Prävalenzen von 6,4 % für Rehwild und 2,4 % für Rotwild [10]. Da positive Nachweise in Wildwiederkäuern vor allem bei gleichzeitig hohen HEV-Prävalenzen in Wildschweinen desselben Gebietes gefunden werden und die detektierten Stämme oft identisch sind, wird hier von „Spillover-Infektionen“ ausgegangen. Für andere Tierarten in Deutschland liegen nur wenige Daten vor. Eine Studie, deren Fokus auf Rindern lag, erbrachte nur negative Resultate [11]. Eine detaillierte Studie an Zootieren zeigte, dass vor allem Schweineartige und Fleischfresser hohe Antikörperprävalenzen aufweisen [12]. Bei den Fleischfressern ist allerdings noch unklar, ob sie auch auf den Menschen übertragbare HEV-Typen beherbergen, bei ihnen werden vor allem spezielle Stämme aus der Spezies *Orthohepevirus C* gefunden.

## Übertragungswege des HEV-Genotyps 3

**Figure 1:**
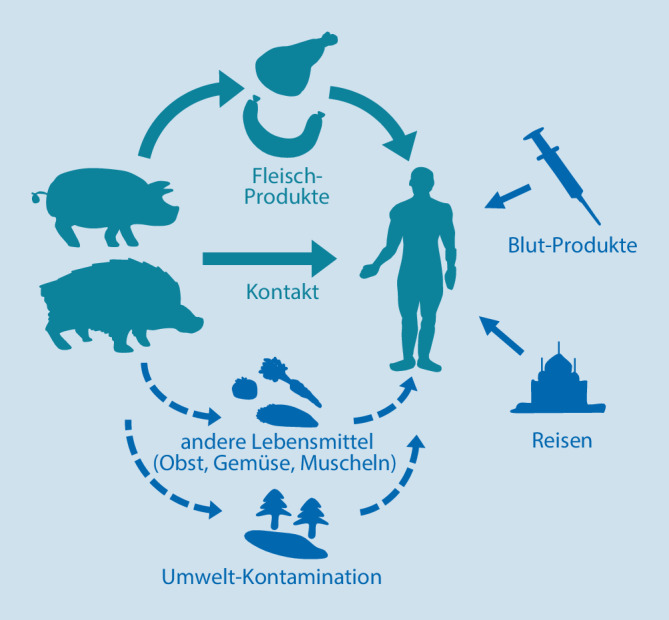

Beim HEV-Genotyp 3 sind verschiedene Übertragungswege bekannt (Abb. 1). Aufgrund der weiten Verbreitung des Virus in Tierreservoiren wurde in verschiedenen Studien unter anderem die Übertragung über direkten Kontakt zu Tieren untersucht. Dies betrifft hauptsächlich serologische Studien, in denen die Antikörperprävalenz von beruflich mit Tieren in Kontakt Stehenden mit der einer Kontrollgruppe verglichen wurde. In den meisten Fällen weisen die Ergebnisse auf ein höheres Infektionsrisiko der tierexponierten Gruppe hin, so beispielsweise bei Wildschweinjägern in Japan mit einer Antikörperprävalenz von 25,5 % im Gegensatz zu 5,5 % bei nichtjagenden Bewohnern derselben Region oder von 30 % bei Arbeitern auf Schweinefarmen in Norwegen im Vergleich zu 14 % bei Blutspendern [6]. Eine deutsche Studie fand eine Antikörperprävalenz von 28,3 % in der Gruppe von beruflich mit Schweinen Arbeitenden im Vergleich zu 15,5 % bei Blutspendern [13]. Eine andere Studie fand bei Waldarbeitern in Deutschland eine Antikörperprävalenz von 18 % im Vergleich zu 11 % bei Blutspendern [14]. Auch wenn hier der konkrete Übertragungsweg unklar ist, könnte der erhöhte Kontakt zu HEV-infizierten Wildtieren und deren Ausscheidungen oder eine häufigere Beteiligung an Jagden die höhere Prävalenz in der Waldarbeitergruppe erklären.

Als ein Hauptübertragungsweg wird der Verzehr von Fleischprodukten, die von infizierten Tieren stammen, angesehen [6]. Dieser Weg wird auch direkt durch Ergebnisse von Fall- und Ausbruchsuntersuchungen, bei denen derselbe Virusstamm sowohl im Patienten als auch in Resten des verzehrten Lebensmittels nachgewiesen wurde, gestützt. So wurden aus Japan verschiedene Erkrankungsfälle nach dem Verzehr von nicht oder ungenügend erhitzter Leber oder Fleisch von Wildschweinen oder Rehwild aufgeklärt. In Frankreich wurde ein Hepatitis-E-Ausbruch mit 7 erkrankten Personen beschrieben, die eine kontaminierte traditionelle Wurst mit rohen Schweineleberanteilen gegessen hatten [15]. Auch aus der Schweiz ist ein ähnlicher Erkrankungsfall aufgeklärt worden, der nach dem Verzehr einer anderen Wurstart mit roher Schweineleber aufgetreten war [16]. Für Deutschland wurden verschiedene Fall-Kontroll-Studien durchgeführt, die auf den Verzehr von Wild und Innereien [17], Schweineleber, Schweine- und Wildschweinfleisch [18] sowie Wurstprodukten [19] als höchsten Risikofaktor für eine Hepatitis-E-Erkrankung hinweisen.

Neben Fleischprodukten wurden verschiedentlich auch Hinweise auf andere Lebensmittel als mögliche Übertragungsvehikel gefunden [20]. Alternative Übertragungswege werden auch von Ernährungsstudien gestützt, in denen Vegetarier regelmäßig auch positive Antikörperbefunde tragen, wenngleich meistens mit deutlich geringerer Prävalenz als Omnivoren. HEV-RNA wurde auch an Beerenfrüchten und in Muscheln nachgewiesen. Für Deutschland gibt es hierzu bisher nur wenige Daten. Als Ursache für die HEV-RNA-Nachweise in diesen Lebensmittelarten werden unter anderem Kontaminationen mit Abwasser oder Dünger aus Schweinehaltungen angesehen.

Umweltkontaminationen mit Ausscheidungen von infizierten Schweinen, Wildtieren und Menschen werden auch als Ausgangspunkt für HEV-Infektionen diskutiert. Eine erste deutsche Studie hat beispielsweise in Abwasser und Flusswasser HEV-RNA nachweisen können [21]. Auch wenn deshalb Infektionen durch Aufnahme des kontaminierten Wassers möglich erscheinen, ist die relative Bedeutung dieses Übertragungsweges noch unklar.

Mensch-zu-Mensch-Übertragungen durch direkten Kontakt scheinen bei HEV und im Besonderen beim HEV-Genotyp 3 kaum vorzukommen. Demgegenüber gelten Blut und Blutprodukte als mögliche Übertragungsvehikel. Studien in Deutschland konnten HEV-RNA in 1 von 4525 bis 1 von 597 der untersuchten Blutspenden nachweisen [22, 23]. Aufgrund des Risikos einer HEV-Übertragung durch Blutprodukte sind seit 2020 Blutspenden in Deutschland auf HEV zu untersuchen [24].

Weiterhin gelten Reisen in Länder, in denen die Genotypen 1 und 2 endemisch sind und aufgrund niedriger Hygienestandards über kontaminiertes Trinkwasser übertragen werden könnten, als Risiko für eine Infektion mit HEV.

## Bedeutung für die Lebensmittelsicherheit

Wie oben bereits ausgeführt wurde, haben Fleisch und Fleischprodukte von Schweinen und Wildschweinen eine besondere Bedeutung für die Übertragung von HEV in Deutschland. In einigen Studien wurde untersucht, wie weit HEV-RNA in solchen Lebensmitteln in Deutschland verbreitet ist (Tab. 3). Für Schweineleber aus dem Handel wurden hierbei Nachweisraten von 4,0–4,9 % gefunden. In Leberwürsten und -paté wurden 12,5–22,0 % RNA-positive Proben gefunden. Bei Rohwürsten ohne Leberanteil (z. B. Salami) ergab eine Studie etwa 20 % positive Proben, während eine andere Studie keine RNA in solchen nachweisen konnte. Die Verwendung unterschiedlicher Methoden mit unterschiedlichen Nachweisgrenzen könnte die Diskrepanz zwischen beiden Studien erklären. Generell scheinen die höheren Nachweisraten für Wurstwaren im Vergleich zum Ausgangsmaterial (z. B. Leber) zunächst überraschend. Jedoch muss in Betracht gezogen werden, dass während der Herstellung meistens Fleisch und Organe mehrerer Tiere gemischt werden, wodurch sich die höheren Nachweisraten erklären lassen.

**Table 3:** 

Lebensmittel	Nachweis von HEV-RNA (Zahl positiver/Gesamtzahl untersuchter Proben)	Referenz
Leber	8/200 (4,0 %)	[45]
	2/41 (4,9 %)	[46]
Leberwurst	11/50 (22,0 %)	[47]
	5/40 (12,5 %)	[46]
Leberpaté	6/40 (15,0 %)	[46]
Rohwurst (ohne Leber)	14/70 (20,0 %)	[47]
	0/10 (0,0 %)	[46]

Bei der Interpretation der Nachweisraten ist generell problematisch, dass, methodisch bedingt, bisher nur RNA-Nachweise erfolgen können. Der Nachweis von infektiösen Viren direkt in Lebensmitteln, beispielsweise durch Anzucht in Zellkulturen, ist wegen des Fehlens entsprechender praktikabler und sensitiver Methoden derzeit noch nicht möglich. Weil aber die meisten Lebensmittel während ihrer Herstellung verschiedene physikalisch-chemische Prozesse durchlaufen, könnte das enthaltene Virus währenddessen inaktiviert worden sein. Deshalb ist die Identifizierung von Risikolebensmitteln zurzeit noch schwierig und mit vielen Unsicherheiten behaftet.

Um Anhaltspunkte für die Stabilität von HEV in speziellen Lebensmitteln und unter spezifischen physikochemischen Bedingungen zu erhalten, wurden sowohl Inokulationsversuche mit Schweinen als auch Zellkulturuntersuchungen durchgeführt. So wurden unterschiedliche Lebensmittel verschiedenen Erhitzungsregimes unterzogen, danach durch intravenöse Injektion der aufgearbeiteten Proben in Schweine inokuliert und anschließend deren Serokonversion gemessen. Hierbei wurde gezeigt, dass eine Erhitzung von 71 °C Kerntemperatur für 5 min bei Leber [25] und von 71 °C für 20 min bei Leberpaté [26] zur HEV-Inaktivierung ausreichend war. In einer Zellkulturstudie führte die Erhitzung von HEV in Zellkulturmedium bei 70 °C für 1 min zu einer 1000-fachen Reduktion der Infektiosität, während bei 60 °C für 1 min nur eine 10-fache Reduktion erreicht wurde [27]. In diesem Zellkultursystem wurden auch andere Bedingungen genauer untersucht, wobei sich generell eine hohe Virusstabilität abzeichnete: Nur eine geringe Verminderung der Infektiosität (≤ 10-fache Reduktion) wurde bei pH-Werten zwischen 2 und 9 für 1 h [28], einer Salzkonzentration von 20 % NaCl für 24 h [29] oder einem hydrostatischen Druck von 200 MPa für 1 min gefunden [30]. Starke Inaktivierungen (> 1000-fache Reduktion) waren nur bei pH 1 und 10 sowie bei 600 MPa für 1 min festzustellen. Insgesamt deuten die Ergebnisse darauf hin, dass HEV in den meisten üblichen Lebensmitteln, die keiner starken Erhitzung oder hohen Drücken ausgesetzt sind, für lange Zeit infektiös bleiben kann.

## Weiterer Forschungsbedarf

Aus den bisherigen Ausführungen kann weiterer Forschungsbedarf abgeleitet werden, um die Übertragung von HEV auf den Menschen und die dadurch ausgelösten Erkrankungsfälle zu verringern. Beim Übertragungsweg über tierische Lebensmittel gilt es zunächst, die Risikoprodukte, die hohe Mengen von infektiösem HEV enthalten können, weiter einzugrenzen. Dies könnte über detaillierte Untersuchungen bei einzelnen Erkrankungsfällen inklusive der Untersuchung von Lebensmittelresten oder über weitere Fall-Kontroll-Studien erfolgen. Auch die Untersuchung des Inaktivierungsverhaltens von HEV während der Herstellung verschiedener Fleischprodukte kann klären helfen, unter welchen Bedingungen HEV vollständig inaktiviert wird und welche Produkte somit kein Risiko darstellen. Hierzu wäre allerdings die vorherige Entwicklung von robust anwendbaren Methoden zur Infektiositätstestung von HEV in Lebensmitteln vonnöten. Wenn die entsprechenden Risikoprodukte identifiziert sind, könnten gezielte Verzehrempfehlungen besonders für Risikopatienten ein sinnvolles Instrument zur Verhinderung von Erkrankungen sein.

Weiterer Forschungsbedarf liegt in der Entwicklung von Strategien zur Minimierung des Kontaminationsrisikos von tierischen Lebensmitteln. Hierfür sollte die gesamte Lebensmittelkette, ausgehend von der Schweinehaltung, genauer untersucht werden. Beispielsweise könnte die Effizienz verschiedener Hygiene‑, Haltungs- und Aufzuchtregimes zur Minimierung von HEV-Infektionen in Schweineherden analysiert werden. Auch die Möglichkeit von Impfungen in den Tierbeständen sollte hierbei genauer untersucht werden. Für die Schlachtung und Lebensmittelproduktion könnten verschiedene Hygienemaßnahmen auf ihre Anfälligkeit für Kreuzkontaminationen mit HEV analysiert werden. Untersuchungen zur Wirksamkeit von Desinfektionsmitteln gegenüber HEV, die auch mögliche Unterschiede zwischen unbehüllten HEV-Partikeln und solchen mit einer zusätzlichen Lipidhülle berücksichtigen, sind für die Entwicklung effizienter Desinfektionsregimes nötig. Außerdem wären Untersuchungen zu wirksamen Behandlungen von Lebensmitteln zur Reduzierung von HEV, zusätzlich zu Temperatur- und Hochdruckbehandlung, sinnvoll.

Wichtig wären auch weitergehende Untersuchungen zu alternativen Übertragungswegen. Besonders die Bedeutung von Umweltkontaminationen für die Übertragung von HEV wäre hier von Interesse. Dabei kämen sowohl den Kontaminationen von Gewässern, Übertragungen über Stäube oder aber Kontaminationen von pflanzlichen Lebensmitteln über Düngemittel oder die Bewässerung infrage. Auch hier ließen sich nach Vorliegen der Daten sicherlich effiziente Strategien zur Verhinderung solcher Kontaminationen erarbeiten.

## Fazit

Die Zahl der gemeldeten Hepatitis-E-Fälle nahm in den letzten Jahren stark zu und chronische Infektionen bei Transplantationspatienten stellen eine relativ neue und ernst zu nehmende Problematik dar. In Deutschland kommt vor allem der zoonotische Genotyp 3 vor. Dieser ist in Haus- und Wildschweinen in Deutschland weitverbreitet und kann durch direkten Kontakt zu den Tieren oder durch den Verzehr daraus hergestellter Lebensmittel übertragen werden. HEV wird auch in verschiedenen Fleischprodukten in Deutschland nachgewiesen, wobei allerdings zumeist unklar ist, ob das nachgewiesene Virus noch infektiös ist oder während der Lebensmittelherstellung bereits inaktiviert wurde. Forschungsbedarf besteht weiterhin bei der Identifizierung von Risikolebensmitteln und bei Strategien zur Minimierung des Kontaminationsrisikos von Lebensmitteln, auch unter Berücksichtigung möglicher Übertragungswege in der Umwelt.

Das Bundesinstitut für Risikobewertung (BfR) hat verschiedene Empfehlungen erarbeitet, um das Risiko einer Ansteckung über die derzeit am besten untersuchten Übertragungswege zu minimieren [31]. So wird Jägern empfohlen, bei der Ausweidung von Wildschweinen generell Handschuhe zu tragen, um eine direkte Virusübertragung auszuschließen. Allgemein wird Verbrauchern empfohlen, Schweine- und Wildschweinfleisch sowie -leber vor dem Verzehr immer gut durchzuerhitzen, um eventuell enthaltenes Virus sicher zu inaktivieren. Für Risikogruppen wie Personen mit Lebervorschädigung oder Transplantationspatienten wird empfohlen, auf den Verzehr von kurzgereiften Rohwürsten zu verzichten, weil durch deren Herstellungsweise möglicherweise enthaltenes Virus wahrscheinlich nicht inaktiviert wird. Die Empfehlungen werden kontinuierlich angepasst, wenn neue belastbare Erkenntnisse zu Übertragungswegen, Risikolebensmitteln oder effizienten Inaktivierungsmethoden vorliegen.
